# A health systems strengthening intervention to improve quality of care for sick and small newborn infants: results from an evaluation in district hospitals in KwaZulu-Natal, South Africa

**DOI:** 10.1186/s12887-019-1396-8

**Published:** 2019-01-24

**Authors:** C. Horwood, L. Haskins, S. Phakathi, N. McKerrow

**Affiliations:** 1 Centre for Rural Health, George Campbell Building, Howard College Campus, University of KwaZulu-Natal, Durban, South Africa; 2 KwaZulu-Natal Department of Health, Pietermaritzburg, South Africa; 3 Department of Paediatrics and Child Health, University of KwaZulu-Natal, Durban, South Africa

**Keywords:** Quality of care, Newborn, Health-system strengthening, South Africa

## Abstract

**Background:**

Many newborn infants die from preventable causes in South Africa, often these deaths occur in district hospitals. A multipronged intervention aiming to improve quality of newborn care in district hospitals was implemented comprising training in clinical care for sick and small newborns, skills development for health managers, on-site mentoring, and hospital accreditation. We present the results of the project evaluation.

**Methods:**

We conducted three sequential cross-sectional surveys in 39 participating district hospitals at baseline, midpoint and endpoint of the three-year intervention period. Data were collected by a trained midwife using a series of checklists including: availability of trained staff, drugs and equipment; newborn care practices; perinatal mortality audits; neonatal unit staff skills; quality of record keeping. A scoring system was developed for three domains: resources; care practices; resuscitation equipment, and a composite score that included all variables measured. Health worker (HW) knowledge was assessed at midpoint and endpoint.

**Results:**

The average score for resources increased from 13.5 at baseline to 22.6 at endpoint (maximum score 34), for care practices from 17.7 to 22.6 (maximum score 29), and for resuscitation equipment from 10.8 to 16.1 (maximum 25). Average composite score improved significantly from 42.0 at baseline to 55.7 at midpoint to 60.7 at endpoint (maximum score 88) (*p* = 0.0012). Among 39 participating hospitals, 38 achieved higher scores at endpoint compared to baseline. Knowledge was higher among HWs trained during the project at midpoint and endpoint. Gaps that remained included poor infrastructure, lack of resuscitation equipment in some areas, poor postnatal care and lack of a dedicated doctor.

**Conclusions:**

This intervention achieved measurable improvements in many important elements contributing to newborn care. A scoring system was used to track progress, compare facilities’ performance, and identify areas for improvement. Various methods were used to generate the quality of care score, including skills assessment and record reviews. However, measuring quality of clinical care and outcomes was challenging, and we were unable to determine whether the intervention improved clinical care and lead directly to improved outcomes for babies. In developing a future score for quality of care, a stronger focus should be placed on assessing clinical care and outcomes.

## Background

The global burden of neonatal mortality is substantial. In 2016 an estimated 7000 newborn babies died each day, with newborn deaths accounting for 46% of all deaths among children under 5 years [1]. Poor intrapartum and newborn care is also associated with a major burden of disability [2]. As child mortality has improved in recent years, achieving improvements in neonatal mortality has fallen behind, as a result of which neonatal deaths make up an increasing proportion of all child deaths. Improving outcomes for newborn babies has, therefore, been identified as a priority for global child health [3]. Proven, cost-effective interventions exist to manage the major causes of deaths in newborns. Key actions which can be implemented at scale in resource constrained settings in order to prevent up to three million global newborn deaths are outlined in the WHO/UNICEF Every Newborn Action Plan [4].

Neonatal deaths occur predominantly in the first week of life, with nearly 50% of deaths occurring in the first 48 h. Effective interventions include improved delivery care, immediate care for the infant at delivery, preventive care for the healthy newborn, as well as care for sick and small newborns. Improving care for small and sick newborns has been neglected in the past, and it is estimated that this could prevent close to 600,000 newborn deaths globally every year, with most of this effect being achievable with district hospital care [5]. Care for small and sick newborns includes extra thermal care, support for feeding, antibiotics for infection, and kangaroo mother care. Improving quality of care for sick and small newborns requires health personnel that are trained and equipped to manage these babies. Inpatient care facilities play a crucial role for newborns requiring full supportive facility care [4]. In particular, all staff members with a role in caring for newborn babies must be trained and competent in newborn resuscitation, since up to 10% of newborns may require stimulation at birth and 5% require resuscitation at birth [6]. Adequate skills and facilities are needed at all levels of the health system if good quality newborn care is to be accessible for all babies.

Child mortality in South Africa (SA) remains unacceptably high with a large proportion of child deaths occurring in the neonatal period [7], so that improving outcomes for these infants is a priority. Between January 2012 and December 2013, more than 14,000 early neonatal deaths were recorded in the SA National Perinatal Problem Identification Programme (PPIP) database from 588 PPIP sites in the country [8]. Many births (46.5%) and neonatal deaths (42.3%) in SA occur in community health centres and district hospitals [8] where clinical services are provided by generalist doctors and nurses. As in other lower resource settings, many newborn deaths in SA are from potentially preventable causes, with the major causes of perinatal deaths being intrapartum birth asphyxia and prematurity. Preventable factors identified by the PPIP programme in 2012–13 included lack of equipment in the neonatal unit, inadequate neonatal management plans, and inadequate monitoring of babies’ condition [8]. All facilities where deliveries occur need appropriate resources and expertise to provide care for these infants, if deaths and disability are to be prevented. Improving newborn care, particularly in district hospitals, is key to the reduction of perinatal and neonatal mortality, and it is estimated that this could prevent thousands of infant deaths in SA.

This paper describes an evaluation of the quality of care provided at 39 district hospitals in KwaZulu-Natal (KZN) province over a three-year period during which a structured, multipronged initiative was undertaken to support newborn care in district hospitals. This initiative was known as the KwaZulu-Natal Initiative for Newborn Care (KINC).

## Methods

An observational, cross-sectional facility survey was conducted in all 39 district hospitals in KZN at baseline, midpoint and endpoint of the 3 year intervention period, using the same data collection tools at each time point. An experienced professional nurse/midwife, who had attended training in the management of sick and small newborns (MSSN), conducted all data collection. At each time point, each facility was visited for a single day to collect data. Hospitals were informed of the date of the visit but were not provided with information about the assessment, and involvement of hospital staff during the visit was minimised. At midpoint and endpoint all staff on duty in the neonatal unit on the day of the visit were requested to complete a self-administered questionnaire to assess knowledge of KZN neonatal care guidelines.

### Study setting

KZN is one of 11 provinces in South Africa and has a population of approximately 11 million people. At the time of the study, there were 39 district hospitals, 10 regional hospitals and one tertiary hospital in KZN providing care to newborn babies. This paper focusses on district hospitals, which provide generalist health services and support to primary health care clinics within a sub-district. These hospitals have between 30 and 300 beds, a 24-h emergency service and an operating theatre. District hospitals are defined by the level of services provided so that, although most district hospitals are small and located in isolated areas, several large urban hospitals are designated as district hospitals. The number of deliveries conducted in participating hospitals varied widely, from 1000 to 6000 per annum, highlighting the heterogeneity among participating hospitals. However, by definition, paediatric care in all district hospitals is provided by generalist medical practitioners, supported by monthly outreach visits from paediatricians from regional or tertiary hospitals. In the neonatal unit, care is provided by a team of nurses under the guidance of an advanced midwife, a nurse with specialist midwifery training that includes neonatal care. High care services, including continuous positive airways pressure (CPAP) but not intensive care, are provided at district hospitals. Intensive care is defined by provision of artificial ventilation, and requires additional supportive resources including staffing, equipment and space. Intensive care is provided at regional referral hospitals, often located several hours away from the district hospitals.

### Description of the KINC intervention

**Fig. 1:**
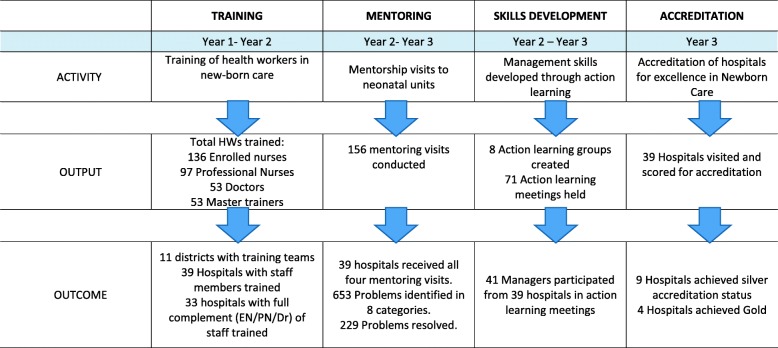
Intervention activities

KINC was a multi-pronged health system strengthening intervention undertaken over a 3 year period (2013–2016), aimed at addressing challenges to provision of high quality newborn care in 39 district hospitals in KZN. A KINC task team was set up to oversee and plan project activities, led by the KZN Department of Health, and including key role-players from all levels of the health system and from all health districts. Ahead of KINC implementation, a two-day orientation workshop was conducted with managers responsible for management of neonatal nurseries in district hospitals to promote buy-in and ensure awareness and support for the initiative. The intervention consisted of implementation of the guidelines for management of small and sick newborn infants (MSSN) in district hospitals developed by the SA Department of Health [9]. MSSN implementation included: training for all cadres of health workers (HWs); development of training teams for MSSN in every district; skills development for health managers using an action learning methodology; and on-site mentoring visits to district hospitals (Fig. 1). A five-day MSSN training was conducted with doctors and nurses from all district hospitals, which included both theory and clinical practice and was conducted at the local referral hospital wherever possible, to build team work between HWs at district and referral hospitals. KINC training focussed primarily on developing knowledge and skills of HWs to manage sick and small newborns. Topics included: assess and classify; treat, observe and care (including maintaining normal body temperature, safe oxygen therapy, maintaining normal glucose, feeds and fluids; infection prevention and control; referral); assess feeding and counsel; follow up; and routine care for all newborns. Management of specific conditions of newborns included: apnoea/respiratory distress; preterm/low birth weight; acute infection; encephalopathy; seizures; jaundice; congenital abnormalities; syphilis; tuberculosis; and HIV. The training package consisted of newborn care chart books; training manuals; exercise manuals and facilitator’s manuals. Drug dosages, charts and recording forms were supplied in the training package. Participants were selected for training using routine DoH systems based on whether they were working in the neonatal nursery.

Mentoring visits were conducted by an advanced midwife, in partnership with district maternal child and women’s health (MCWH) supervisors, using a structured mentoring tool. Mentoring activities included assessment of: availability and functionality of essential newborn care equipment; availability of personnel in the neonatal nursery; compliance with infection prevention and control practises, and auditing of newborn records to assess compliance with MSSN guidelines. On completion of the visit challenges identified were presented to senior hospital and district management for their attention. Proposed actions were reviewed on subsequent visits.

On completion of the intervention, accreditation visits were conducted to all district hospitals by a team of senior clinicians and managers to assess the hospital’s performance, and to determine whether each hospital had achieved accreditation status for excellence in newborn care. Activities included in the accreditation assessment and accreditation outcomes for district hospitals, are described elsewhere [10].

### Measurements

A series of structured observation checklists, based on KZN Department of Health (DoH) norms and standards for newborn care, were developed to collect data in each facility. Items observed included staffing, infrastructure, availability and deployment of equipment, consumables, resuscitation equipment, availability of guidelines and policies, and compliance with required audit practices. Assessment tools were developed in consultation with specialist neonatologists in KZN, and were designed to be valid and reliable when used by a data collector with basic skills in neonatal care. Wherever possible these items were determined by direct observation, however where this was not possible staff members were asked.

In addition, staff members on duty were requested to demonstrate skills and care practices in the neonatal nursery and their performance was recorded on a checklist. Staff members were selected according to availability and convenience. The skills topics included were as follows: checking an ambubag is working correctly; changing incubator temperature appropriately; assembling a CPAP circuit: calculating fluids and setting up an infusion pump correctly. These topics were selected as being critical skills required for basic nursery function, where the correct response can be clearly defined.

Ahead of the visit, staff on the neonatal nursery were requested to provide the clinical records of the five babies most recently discharged from the nursery. A review of these records was conducted to determine the quality of record keeping.

At midpoint and endpoint, a self-administered questionnaire was used to test knowledge of neonatal care practises among all health workers, including doctors and other cadres of health workers in the neonatal nursery on the day of the visit. The knowledge questionnaire comprised a total of 33 questions, eight true/false questions and 25 multiple-choice questions.

### Data analysis

Scores were developed to provide an assessment of quality of care provided, based on compliance with relevant norms and standards using IBM SPSS Statistics 24.0 (IBM Corporation, Armonk, NY, USA), and were split into three domains. Firstly, a *Resources Score,* comprising 34 items, was developed to measure compliance with staffing; equipment; infrastructure, and consumables (Table 1). Secondly a *Care Practices Score*, comprising 19 care indicators plus the results of five record reviews, was developed to measure compliance with admission policies; monitoring and evaluation activities; appropriately deployed equipment; ability to use the equipment; kangaroo mother care; postnatal care (Table 2). The record review was scored out of 10, based on whether the following data elements were recorded: date of birth (1/2); date of admission (1/2); baby’s weight daily (1); 3-hourly observations for first 24 h (1); 3- hourly blood glucose levels for first 24 h (1); mothers HIV status (1); mothers RPR result (1); gestational age of the baby (1); daily doctors ward rounds (2), and outcome either death/referral/discharge (1). If the staff were unable to produce the clinical records of the five most recent discharged babies, missing records scored zero. Scores for each of the five records were averaged to give an overall score for each hospital which was added to the care practices score. Clinical skills were assessed by requesting staff to undertake a clinical activity while being observed. This was scored as 1 = correct or 0 = incorrect.

**Table 1:** Compliance with items contributing to the Resources Score (34 items)

	KZN Norm	Data source	All district hospitals		
			Compliant at baseline *n* = 39 (%)	Compliant at midpoint *n* = 38 (%)	Compliant at endpoint *N* = 39 (%)
HUMAN RESOURCES					
Dedicated doctor responsible for the nursery	*recommended*	Observation or staff report	12 (31%)	34 (89%)	39 (100%)
Dedicated doctor has neonatal training (MSSN training)	*recommended*	staff report	5 (12.8%)	22 (57.9%)	10 (25.6%)
Registered nurse on duty with neonatal/MSSN training	*recommended*	staff report	15 (38.5%)	33 (86.8%)	31 (79.5%)
INFRASTRUCTURE					
Stand-alone nursery (separate door not part of labour ward)	*recommended*	Observation	36 (92.3%)	38 (100%)	39 (100%)
Wall oxygen points	*2 per HC bed* *1 per IC bed*	Observation	6 (15%)	3 (8%)	7 (18%)
Functioning portable oxygen points	*1 per nursery*	Observation	14 (36%)	17 (45%)	22 (56%)
Wall suction points	*2 per HC bed* *1 per IC bed*	Observation	2 (5%)	3 (8%)	8 (21%)
Functioning portable suction	*1 per nursery*	Observation	31 (79%)	26 (68%)	31 (80%)
Functioning electrical points	*12 per HC bed*	Observation	1 (3%)	5 (13%)	3 (7.7%)
Medical air or compressor	*1 per nursery*	Observation	15 (38%)	13 (34%)	22 (56%)
MAJOR EQUIPMENT					
Functional CPAP machine available	*1 per nursery*	Observation	8 (21%)	12 (32%)	14 (36%)
Multiparameter monitors	*1 per HC bed*	Observation	6 (15%)	21 (55%)	28 (72%)
Oxygen blenders	*1 per HC bed*	Observation	1 (3%)	2 (5%)	7 (18%)
Glucometers	*1 per cubicle*	Observation	33 (85%)	35 (92%)	37 (95%)
Stethoscopes	*1 per HC bed*	Observation	5 (13%)	21 (55%)	26 (67%)
Infusion pumps	*2 per HC bed*	Observation	8 (21%)	9 (24%)	17 (44%)
Syringe drivers	*4 per HC bed*	Observation	0 (0%)	1 (3%)	2 (5%)
CONSUMABLES/DRUGS					
CPAP circuits	*1 complete set* ^*a*^	Observation	13 (33.3%)	17 (44.7%)	15 (38.5%)
Neonatal nasal prongs	5^a^	Observation	23 (59%)	29 (76%)	36 (92%)
Oxygen tubing	5^a^	Observation	16 (41%)	25 (65.8%)	22 (56%)
Infusion sets	10^a^	Observation	19 (49%)	18 (47%)	29 (74%)
Pulse oximeter probes	HC beds + 1^a^	Observation	32 (82%)	36 (91%)	39 (100%)
Gentamicin	any^a^	Observation	32 (82%)	35 (92%)	36 (92%)
Pen G	any^a^	Observation	27 (70%)	31 (82%)	32 (82%)
Neonatalyte	200mls X 10^a^	Observation	15 (38%)	26 (68%)	27 (69%)
Glucostix	10^a^	Observation	34 (87%)	36 (95%)	35 (90%)
Neonatal cannulae	20^a^	Observation	29 (74.4%)	34 (89%)	37 (95%)
AVAILABILITY OF GUIDELINES IN THE NEONATAL UNIT					
MSSN chart booklet	*recommended*	Observation	23 (59%)	35 (92%)	37 (95%)
Fluids chart on the wall	*recommended*	Observation	14 (36%)	22 (58%)	34 (87%)
Resuscitation chart on the wall	*recommended*	Observation	8 (21%)	17 (45%)	27 (69%)
Feeding chart on the wall	*recommended*	Observation	12 (31%)	20 (53%)	34 (87%)
Incubator temperature chart	*recommended*	Observation	8 (21%)	19 (50%)	29 (74%)
Phototherapy chart on the wall	*recommended*	Observation	25 (64%)	32 (84%)	39 (100%)
Oxygen chart on the wall	*recommended*	Observation	5 (13%)	8 (21%)	9 (23%)
OVERALL RESOURCES SCORE/34					
Average score (highest-lowest)			13.5 (5.0–26)	19.4 (11.0–28)	22.1 (11.0–31.0)

^a^indicates the number of items that had to be present to achieve compliance

**Table 2:** Compliance with items contributing to the care practices score (29 items)

19 items plus 10 points for record review		All district hospitals		
	Data source	Baseline N = 39 (%)	Midpoint N = 38 (%)	End point N = 39 (%)
All sick neonatal admissions admitted to nursery	Observation or staff report	20 (51%)	29 (76%)	29 (74%)
CS babies not routinely admitted to nursery	Observation or staff report	30 (77%)	33 (87%)	36 (92%)
CLINICAL AUDIT				
PPIP being implemented	Observation or staff report	37 (95%)	35 (92%)	36 (92%)
3 months PPIP data available	Observation	27 (69%)	33 (87%)	32 (82%)
Perinatal review meeting minutes available for past three months	Observation	23 (59%)	32 (84%)	33 (85%)
KANGAROO MOTHER CARE				
No of KMC beds compliant with recommended number KMC beds	Observation	26 (67%)	34 (90%)	36 (92%)
POSTNATAL WARD				
Babies kept with the mother at all times	Observation	37 (95%)	37 (97%)	36 (92%)
Observations done 12 hourly on babies born by caesarean section	Observation	9 (23%)	14 (37%)	9 (23%)
Daily weights recorded on all babies	Observation/ review of clinical notes	8 (21%)	13 (34%)	11 (28%)
Babies are not routinely bathed (recommended)	Observation or staff report	16 (41.0%)	37 (97.4%)	35 (98.7%)
Bottles and teats NOT on view in the postnatal ward (recommended)	Observation	23 (59.0%)	36 (94.7%)	31 (79.5%)
Road to health cards are available	Observation	36 (92.3%)	36 (94.7%)	36 (92.3%)
STAFF PRACTICES				
Able to check ambubag	Observation	15 (38%)	31 (82%)	34 (87%)
Able to set incubator temperature	Observation	35 (90%)	34 (90%)	30 (77%)
Able to change incubator temperature according to babies temperature	Observation	33 (85%)	36 (95%)	36 (95%)
Able to assemble CPAP circuit	Observation	6 (15%)	12 (32%)	13 (33%)
*If baby weighs 1.5 kg:*				
Able to calculate fluid requirements on first day of life	Observation	14 (36%)	17 (45%)	31 (80%)
Able to calculate the number of mls/hr	Observation	12 (31%)	20 (53%)	31 (80%)
Able to set up an infusion pump correctly	Observation	25 (64%)	33 (87%)	38 (97%)
OVERALL CARE PRATICES SCORE/29				
Average score (highest –lowest)		17.7 (11–22)	21.6 (16–27)	22.6 (17–27)

Finally, a *Resuscitation Score* was developed to measure availability of essential resuscitation equipment in each relevant clinical area: neonatal unit; operating theatre; labour ward; postnatal ward as shown in Table 3.

**Table 3:** Compliance with resuscitation score items (25 items)

		All district hospitals		
	Data source	Baseline N = 39 (%)	Midpoint N = 38 (%)	End point N = 39 (%)
Neonatal nursery				
Neonatal ambubag & mask functioning	Observation	32 (82.1%)	35 (92.1%)	37 (94.9%)
Correct mask sizes for ambubag (0; 00; 000)	Observation	4 (10.3%)	11 (28.9%)	2 (5.1%)
Laryngoscope set out and working	Observation	22 (56.4%)	30 (78.9%)	32 (82.1%)
Spare batteries for laryngoscope/alternative laryngoscope	Observation	17 (43.6%)	21 (55.3%)	33 (84.6%)
Endotracheal tubes (Sizes 2.5; 3.0; 3.5)	Observation	19 (48.7%)	23 (60.5%)	29 (74.4%)
Adrenaline	Observation	34 (87.2%)	34 (89.5%)	39 (100%)
Labour ward				
Neonatal ambubag & mask functioning	Observation	32 (82.1%)	33 (86.8%)	38 (97.4%)
Correct mask sizes for ambubag (0; 00; 000)	Observation	4 (10.3%)	5 (13.2%)	39 (100%)
Laryngoscope set out and working	Observation	28 (71.8%)	24 (63.2%)	33 (84.6%)
Spare batteries for Laryngoscope/alternative laryngoscope	Observation	16 (41.0%)	22 (57.9%)	30 (76.9%)
Endotracheal tubes (Sizes 2.5; 3.0; 3.5)	Observation	12 (30.8%)	22 (57.9%)	29 (74.4%)
Adrenaline	Observation	33 (84.6%)	36 (94.7%)	36 (92.3%)
Operating theatre (caesarean sections)				
Resuscitaire with heat and light working	Observation	21 (53.8%)	24 (63.2%)	26 (66.7%)
Neonatal ambubag & mask functioning	Observation	31 (79.5%)	36 (94.7%)	34 (87.2%)
Correct mask sizes for ambubag (0; 00; 000)	Observation	2 (5.1%)	4 (10.5%)	2 (5.1%)
Laryngoscope set out and working	Observation	27 (69.2%)	29 (76.3%)	34 (87.2%)
Spare batteries for Laryngoscope/alternative laryngoscope	Observation	16 (41.0%)	18 (47.4%)	31 (79.5%)
Endotracheal tubes (Sizes 2.5; 3.0; 3.5)	Observation	14 (35.9%)	22 (57.9%)	30 (76.9%)
Adrenaline	Observation	28 (71.8%)	35 (92.1%)	36 (92.3%)
Postnatal ward				
Neonatal ambubag & mask functioning	Observation	4 (10.3%)	12 (31.6%)	14 (35.9%)
Correct mask sizes for ambubag (0; 00; 000)	Observation	1 (2.6%)	1 (2.6%)	2 (5.1%)
Laryngoscope set out and working	Observation	6 (15.4%)	10 (26.3%)	11 (28.2%)
Spare batteries for Laryngoscope/alternative laryngoscope	Observation	3 (7.7%)	4 (10.5%)	8 (20.5%)
Endotracheal tubes (Sizes 2.5; 3.0; 3.5)	Observation	3 (7.7%)	8 (21.1%)	13 (33.3%)
Adrenaline	Observation	5 (12.8%)	13 (34.2%)	14 (35.9%)
OVERALL RESUSCITATION SCORE/25				
Average score (lowest – highest)		10.8 (5.0–22.0)	14.4 (7.0–22.0)	16.1 (9–23.5)

All 88 variables were combined to calculate an overall score for each hospital. The Wilcoxon Signed Rank test was used to test the difference between the mean hospital score at baseline and at endline.

The knowledge questionnaire comprised 33 items, each correct answer scored one point, and knowledge scores are presented as the number of correct answers. The non-parametric Mann Whitney U test was used to test differences in the knowledge score for KINC trained and non-KINC trained participants. All significance testing was undertaken using Stata 14 (StataCorp. 2015. Stata Statistical Software: Release 15. College Station, TX: StataCorp LLC.)

## Results

Data was collected in all 39 hospitals at baseline (July–October 2013), and at the endpoint (January–April 2015) and in 38/39 hospitals at midpoint (October–December 2014). The neonatal unit at one district hospital was closed for renovations during the midpoint evaluation.

### Scores

Table 1 shows the compliance with elements contributing to the resources score at each time point.

In generating record review scores (Table 2), records from the five infants most recently discharged from the neonatal nursery were requested from each hospital at each time point. A total of 558 records were reviewed: 179 at baseline; 189 at midpoint; and 190 at endpoint. Of the 39 hospitals, the full complement of all five records were *unavailable* in seven hospitals at baseline, one hospital at midpoint and two hospitals at endpoint. Average score for record reviews at different time points (lowest to highest scores) were as follows: baseline 6.5 (1.5–9.0); midpoint 7.2 (3.6–9.6); endpoint 7.7 (2.1–10.0). Table 2 shows the compliance with elements contributing to the care practices score at each time point.

Mean scores for resuscitation equipment are shown in Table 3. Out of 39 participating hospitals, 36 hospitals (92.3%) showed improvement in the resuscitation scores between baseline and endpoint.

### Average scores

**Fig. 2:**
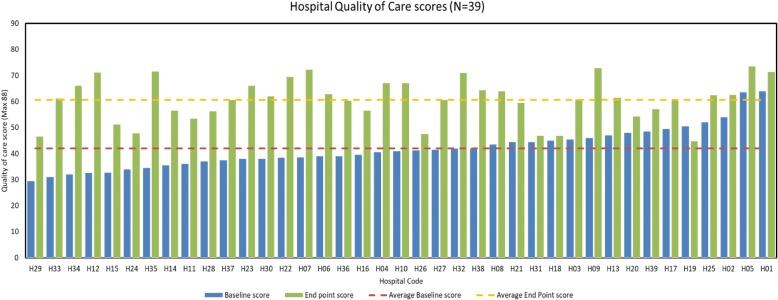
Total quality of care scores

Average scores for resources, care practices and resuscitation equipment from baseline to endpoint are shown in Tables 1, 2, 3. Of the 39 participating hospitals, 35 hospitals (89.7%) scored higher for resources, 33 (84.6%) hospitals scored higher for care practices, and 34 (87.2%) hospitals scored higher for resuscitation at endpoint compared to baseline. There was an overall increase of 62.9% in the average resources score, 26.2% improvement in care practices score, and a 49.1% improvement in resuscitation score from baseline to endpoint. Scores for the composite quality of care score, comprising all 88 items, improved from 42.0 (lowest- highest 29.0–64.0) at baseline to 60.7 (44.8–73.5) at endline, an increase of + 44.5%. There was a significant improvement in total mean scores between baseline and endline (*p* = 0.0012). Individual hospital composite scores at baseline and endpoint are shown in Fig. 2.

### Knowledge of health workers

All health workers on duty on the day of the assessment completed a knowledge questionnaire at midpoint and endpoint. There were 106 health professionals from the 38 district hospitals working in neonatal units on the day of data collection at midpoint (range: 1 to 8 per facility), and 120 health workers in 39 hospitals at endpoint (range: 1 to 6 per facility).

**Fig. 3:**
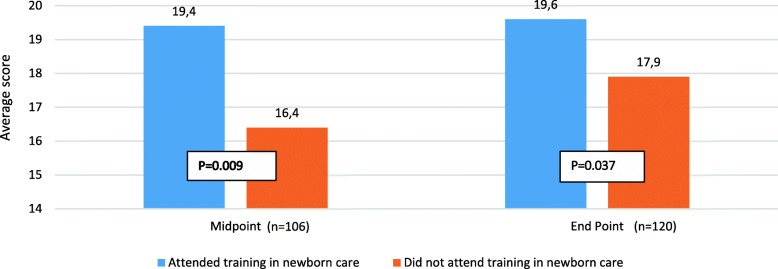
Knowledge scores at midpoint and endpoint

Health workers who had received MSSN training had on average a higher knowledge score than health workers who had not been trained in newborn care over the project period. This was shown at midpoint and was sustained at end point although no further training was conducted between those time points (Fig. 3). Results of the knowledge scores did not contribute to the overall quality of care score.

## Discussion

Our data suggests that a multipronged intervention approach comprising training, mentoring, action learning and accreditation, implemented at scale, can lead to demonstrable improvements to many elements contributing to the quality of care provided for sick and small newborn infants, over a relatively short time period. Improvements were seen in the resources available in most district hospitals, including capital equipment, consumables and drugs, as well as functioning resuscitation equipment and deployment of staff. Such resources are the foundation of providing quality care, without which this cannot be achieved. In addition, care practices also improved, including staff knowledge, observed clinical practices, record keeping and audit practices. Improvements in knowledge scores suggest that HWs trained in newborn care during the project period had a better knowledge of care practices for sick and small newborns, although our methodology does not allow us to clearly infer that KINC training led directly to improved knowledge.

It has been frequently stated that training alone does not lead to sustained change in practice [11], our findings suggest that when combined with mentoring and accreditation, significant improvements can be achieved. This interlinked multipronged approach led to overall strengthening of the health system. For example, training provided neonatal unit staff with information about the newborn care policies that should be followed and why, what equipment was required, and how this equipment should be used. Although procurement of equipment and consumables was not a direct function of this project, during mentoring visits we encouraged health staff to obtain required equipment, and deploy it in the neonatal unit. Progress with deploying equipment was reviewed at subsequent mentoring visits. In this way, we were able to facilitate the transfer of knowledge and skills acquired during training to the workplace. Preparation for accreditation in the final year of the project further reinforced these messages, as hospital managers were informed of the standards against which they would be assessed. During accreditation, each hospital was visited and assessed by a group of senior managers and clinicians, this served to strongly motivate hospital managers to comply with recommendations for their hospital to achieve a good accreditation score [10].

Several challenges were experienced in project implementation including: being unable to train HWs together as hospital teams because of conflicting clinical commitments; patchy coverage of paediatric outreach services and poor buy-in from outreach paediatricians; difficulties with scheduling mentoring visits when all local and district roleplayers were available to participate. We were able to adapt implementation plans to effectively address most problems because of strong leadership from the DoH at provincial and district levels. The provincial KINC task team included role-players from all over the province and meetings were consistently well attended, this allowed child health managers to participate and engage in all decision-making about project activities.

However, key gaps remained, some aspects of infrastructure did not improve, which is to be expected since infrastructure is difficult to change in a short time and requires considerable resources. However, many neonatal units still did not have a designated doctor, aspects of routine postnatal care remained poor, and, despite improvements, essential resuscitation equipment was still not available in all areas at endpoint. The Department of Health failed to provide all the required equipment for CPAP, and as a result implementation of CPAP remained inadequate in most hospitals, despite this having been identified as a key national priority to improve mortality in district hospitals [12]. This highlights the particular complexity of improving care for sick newborn babies. In contrast to many other child survival interventions, significant and ongoing technical expertise and equipment are required to support newborn care. In settings where health workers are scarce and systems for procurement and equipment maintenance are challenging, improvements to resources may be difficult to sustain. We were unable to assess sustainability beyond the completion of the project.

Training in newborn care requires skilled and experienced facilitators, including paediatricians, to teach clinical skills, making ongoing training difficult to sustain in low resource settings. High rates of staff turnover are an additional challenge. A mentorship-based approach could provide an alternative to residential training, so that nurses providing clinical care for newborns in district hospitals could spend time in regional hospitals being mentored and building their skills. Ongoing outreach programmes have a role to play, and outreach paediatricians should view mentoring of neonatal unit staff as a core activity during their visits. Innovative solutions may be required to reduce staff turnover and attract staff to work in neonatal units. More task shifting to nurses, supported by increases in the scope of practice, could be way to achieve this and has been shown to improve care and improve the motivation and retention of nurses [13, 14]. Other approaches could include improved remuneration of nurses with special skills in newborn care, although such interventions must be implemented with care as they may have the unintended consequence of diverting staff from other key areas of practice. Such approaches are identified as applicable to address global challenges in improving motivation of staff and retaining staff working in newborn care [15].

The scoring system developed for this evaluation provided an objective approach to track changes over time in each hospital, compare hospitals at each time point, and identify common shortfalls to prioritise for intervention [16, 17]. However, defining and measuring quality of care is complex [18–20] and multi-dimensional, and different aims for measuring quality may be as diverse as cost containment and patient satisfaction [21]. The most important limitation to our approach was the difficulty in measuring the quality of clinical care, particularly adherence to evidence-based clinical guidelines, which is the most important aspect of providing good quality care. Availability of equipment, consumables and human resources, and even staff knowledge and skills, are relatively easy to measure but do not go far in determining whether the care for newborn infants has actually changed. Although we included a record review to assess quality of care, the complexity of neonatal care for babies whose clinical condition may be varied and unpredictable, made it difficult to determine whether ongoing care was provided according to the guidelines, As a result, care variables were limited to a small number that could be easily assessed, focussing mainly on record keeping and routine observations. We were unable to directly measure adherence to guidelines, this would have required a skilled clinician, which was not feasible for this study.

To address this concern we also considered a number of outcome measures as possible indicators of quality of care, including length of hospital stay, adherence to guidelines and overall in-hospital mortality. However, it was difficult to compare outcomes across different facilities because of the range of clinical conditions and complications that can arise, as well as the substantial differences in numbers of admissions and access to referral care among facilities. In addition, outcomes for newborns are influenced by factors not directly related to clinical newborn care, for example the mother’s socioeconomic situation. All of these factors made it difficult to develop valid and reliable tools to assess and compare clinical care across facilities. We, therefore, acknowledge that while the KINC approach clearly demonstrated improvements in many of the building blocks required for quality care provision, without which this cannot be achieved, we were unable to directly assess whether clinical care or health outcomes improved. However, process indicators have a place, and should remain central to any assessment of quality of care for several reasons: they are easier to measure on an ongoing basis; they allow direct comparison between facilities; they can be measured at a specific time point without having to wait for complicated analysis; and can quickly provide direction for action to address problems. In contrast, addressing poor outcomes requires going back to process indicators to try and explain the poor outcomes [22].

Another challenge was the difficulty of weighting variables to provide a score of quality of care that accurately reflects quality. While it is clear that not all variables are equally important, it was challenging to determine exactly how much more important one element of care was compared to another. We, therefore, chose to use a large number of equally weighted variables to give an overview of quality of care. However, it should be acknowledged that using this approach hospitals could receive good and improving scores, while still failing to comply with key indicators, giving a misleading impression of the quality of care provided. Alternative approaches could be to select several critical indicators and weight these within the scoring system, or penalise hospitals who fail to comply with them. Such an approach worked well in the accreditation process undertaken during this project which is described elsewhere [10].

A future scoring system for quality of care could include additional data elements to strengthen the assessment of quality of care, including a more comprehensive and structured skills assessment for staff, particularly of resuscitation skills in different sites where resuscitation may be required. Vignettes have been successfully used to assess clinical skills in newborn care practices [23], and would have strengthened the methodology in this study. Mothers of infants in the neonatal unit and postnatal ward could also be interviewed to evaluate satisfaction with the care that they and their infants received. A strength of this scoring system was that it was easy to administer, and although a more comprehensive record review and skills assessment would be valuable, this would require skilled assessors and may have compromised the validity and reliability of the tool.

In addition, there were further limitations to the methodology in this study. It is not possible to clearly infer that knowledge scores had increased as a direct result of the intervention because there are alternative explanations for this, including that more competent HWs were selected for training or that those who were trained were more likely to be retained in the nursery and gain further skills. Further, although the data collector was requested to randomly select participants for the skills assessment, this was not done consistently, and it is possible that more skilled HWs were selected for the skills assessment. Finally, the quality of care scores relied on observations and reported findings on a particular day, so that both reporting and observation bias may have led to higher scores being achieved. Overall, it is not possible to draw a clear inference that the improvements demonstrated are directly attributable to the implementation of the KINC programme. However, it was not feasible or acceptable to exclude facilities from the intervention to provide a comparison group, and there was no other initiative directly targeting quality of newborn care over the 3 year KINC implementation period. Although neonatal mortality was not assessed as part of the quality of care score, routine statistics on neonatal mortality in KZN show no trends towards improvement over the period of KINC implementation, but these data are difficult to interpret because of poor quality and incomplete data [24].

Overall, the key to achieving sustained improvement in newborn care is leadership at all levels of the health system, and holding local managers accountable if improvement are not made. This project was strongly supported by current DoH policies, senior managers at the DoH, and district level managers, and was guided by a task team comprising role-players at all levels of the health system, so that extensive buy-in and support was created for improving newborn care. We believe this was pivotal to achieving success.

## Conclusions

In conclusion, combining skills development, with mentoring and accreditation can provide policy-makers with information about gaps in care, where to prioritise additional resources and directly improve quality of care, for newborn infants.

## Abbreviations

CPAP: Continuous Positive Airways Pressure
DOH: Department of Health
EN: Enrolled Nurse
HW: Health worker
KINC: KwaZulu-Natal Initiative for Newborn Care
KMC: Kangaroo Mother Care
KZN: KwaZulu-Natal
MSSN: Management of small and sick newborns
PN: Professional Nurse
PPIP: Perinatal Problem Identification Programme
QOC: Quality of Care
SA: South Africa
UNICEF: United Nations International Children’s Fund
WHO: World Health Organisation
